# Cover

**DOI:** 10.4269/ajtmh.1113_scover

**Published:** 2024-09-03

## Abstract

Clockwise, from top left:

Portrait of President Jimmy Carter and Mrs. Rosalynn Carter, co-founders of The Carter Center, taken at their home in Plains, Georgia. *Photo Credit*: Peter Frank Edwards/Redux, 2013

A South Sudanese child practices using a pipe filter to drink water, thus preventing the spread of Guinea worm disease. The Carter Center leads the international coalition to eradicate the parasitic disease. *Photo Credit*: Louise Gubb/The Carter Center, 2013, Eastern Equatorial State, South Sudan

In Guatemala, a health worker measures a child for proper treatment dosing to prevent river blindness, or onchocerciasis. Guatemala has since eliminated the disease with assistance from The Carter Center and other partners. *Photo Credit*: Peter DiCampo/The Carter Center, 2009, Union Victoria, Guatemala

New Liberian clinicians celebrate their graduation from a training program in child and adolescent mental health, developed by The Carter Center in partnership with the Liberia Ministry of Health, Ministry of Education, and the Ministry of Gender, Children, and Social Protection. *Photo Credit*: Ashoka Mukpo/The Carter Center, 2017, Kakata City, Liberia

At a primary school in Ethiopia, a child washes his face to help prevent the spread of trachoma, a bacterial eye disease. The Carter Center fights trachoma in four African countries: Ethiopia, Niger, Sudan, and South Sudan. *Photo Credit*: William Vazquez/Pfizer, 2013, Ethiopia

